# Management of complications in glaucoma surgery

**DOI:** 10.4103/0301-4738.73689

**Published:** 2011-01

**Authors:** Lingam Vijaya, Panday Manish, George Ronnie, B Shantha

**Affiliations:** Smt. Jadhavbai Nathmal Singhvee Glaucoma Services, Sankara Nethralaya, 18 College Road, Chennai-600006, India

**Keywords:** Blebitis, encysted bleb, leaking bleb, needling, trabeculectomy complications

## Abstract

Surgical option for glaucoma is considered when other modalities are not working out to keep the intraocular pressure under control. Since the surgical procedures for glaucoma disrupt the integrity of the globe, they are known to produce various complications. Some of those complications can be vision-threatening. To minimize the morbidity, it is very important that one should know how to prevent them, recognize them and treat them. The objective of this article is to provide insight into some of those complications that will help the ophthalmologists in treating glaucoma patients in their clinical practice.

Surgical therapy for glaucoma is usually resorted to in case of failure of conservative measures. The first surgical procedure for glaucoma was described by Wecker in 1882 who named it a “filtering cicatrix”.[1] Since then, many different filtering procedures have been attempted to create a fistula between the anterior chamber and the subconjunctival space. Cairns described trabeculectomy in 1968 and reported good results in a series of glaucoma patients.[2] Trabeculectomy was the first guarded filtering procedure and became the most commonly performed surgical procedure. To date, the gold standard surgery for glaucoma is trabeculectomy, because it reduced the frequency of postoperative complications that were associated with full-thickness procedures. Wound healing at the conjunctival and episcleral plane is a limiting factor for the success of trabeculectomy. To counter this, antifibrotics such as 5-Fluorouracil (5-FU)[3] and Mitomycin C (MMC),[4] have been widely used for wound modulation to improve the surgical success.[5] While antifibrotics have improved surgical success, this has come at the cost of increased postoperative complications.[5] Non-penetrating glaucoma surgeries were introduced to minimize the number of glaucoma surgery-related complications.[6] Aqueous drainage implants were initially introduced for refractory glaucomas; however, recently there has been a trend to use the implants with the aim of attempting to avoid trabeculectomy-related complications.[7] In this review we will summarize some of the complications and their management, related to trabeculectomy, non-penetrating glaucoma surgery and aqueous drainage implants.

## Complications with Trabeculectomy

Trabeculectomy provides a non-physiologic route for aqueous outflow and complications may occur despite the best efforts of the surgeon. Timely detection and management of these complications is vital for a good surgical outcome. These complications can be divided into peroperative and postoperative categories.

## Peroperative Complications

Most of the postoperative complications of trabeculectomy are related to peroperative problems involving the conjunctival flap, scleral flap and inner block removal.[8] In this section we will be describing peroperative complications beginning with anesthesia and then proceed through each step of the surgery.

### Anesthesia-related

Generally glaucoma surgery is done under local anesthesia. It is advisable to stop anticoagulant therapy preoperatively to minimize the hemorrhagic complications.[9] Acute retrobulbar hemorrhage presents as proptosis associated with hardening of the eye, discoloration of lids and subconjunctival hemorrhage following local anesthetic injection.[10] Elevated intraocular pressure (IOP) that occurs with retrobulbar hemorrhage can compromise blood flow to the optic nerve in advanced glaucoma. In this situation immediate measures include intravenous administration of mannitol (1 g/kg body weight of 20% solution) and lateral canthotomy with lateral cantholysis. Surgery should be deferred till the complete absorption of hemorrhage. To avoid anesthesia-related complications, topical anesthesia is an option. In a comparative study of topical and retrobulbar anesthesia for trabeculectomy, both procedures provided equally efficacious optimal operative conditions for surgeon and excellent pain control for the patient.[11] In another study lidocaine 2% jelly was compared with sub-Tenon’s anesthesia for trabeculectomy and it was found that patient comfort and surgeon satisfaction was similar in both groups.[12]

### Conjunctival buttonholes

Tears or buttonholes in the conjunctiva and Tenon’s capsule are complications, the consequences of which are far greater than their size. They are difficult to treat and responsible for the failure of the surgery. The main reasons for their occurrence are poor visualization and use of inappropriate instruments. It is of utmost importance to handle the conjunctiva gently using non-toothed forceps. A limbus-based or a fornix-based flap is fashioned according to the surgeon’s preference. Limbus-based trabeculectomy gives the advantage of a watertight closure; on the contrary, leaks are very common with a fornix-based flap.[13] However, the technique of creating a limbus-based flap requires the creation of a conjunctival incision as posteriorly as possible; this makes the conjunctiva susceptible to tears. The fornix-based flap provides a better surgical view and exposure.[14] The important step in the management of conjunctival buttonhole is immediate identification and management. The risk of inadvertent buttonholes is greatest in previously operated eyes that have extensive subconjunctival scarring. Efforts must be taken to avoid the cut edges of the conjunctiva touching the antifibrotic agent used. If the tear is large and satisfactory watertight closure appears difficult, a new surgical site can be chosen. Direct repair of the buttonhole can be performed using a purse-string suture or single or multiple mattress sutures using a 10-0 Nylon suture on an atraumatic needle. All the conjunctival leaks should be closed before concluding the surgery. They may lead to hypotony, shallow anterior chamber (AC) and scarring of the bleb. Early postoperative wound leak was found to be a risk factor for failure of trabeculectomy in the fluorouracil filtering surgery study (FFSS).[15] The surgeon should also test for wound leaks during the immediate postoperative period and manage them promptly.

### Scleral flap complications

The important question the surgeon faces in trabeculectomy is how thick the scleral flap should be. Too thin or thick a flap can both produce complications. An adequate scleral flap depth during dissection is necessary to avoid tearing the flap in superficial dissection or premature entry in deep dissections. In case the flap tears, it may be sutured to the anterior limbal tissue and a new flap created more posteriorly and in a deeper plane. If this is not possible a new flap needs to be fashioned at another site. One must be aware that scleral flaps are difficult to repair in a predictable fashion. In case of total flap amputations at the only available site, we may need to have donor scleral flap reinforcement or a glaucoma drainage device may become necessary. Special care should be exercised in fashioning the flap in buphthalmic and highly myopic eyes. Visualization of dark uveal tissue through the scleral bed indicates very thin underlying sclera. Deeper dissections are prone to posterior premature entry. In these cases, balanced salt solution (BSS) or viscoelastic is injected to deepen the anterior chamber and a superficial lamella is then dissected anteriorly into clear cornea. Other than the thickness, flap size seems to be an important parameter; a large flap seems to produce more diffuse blebs. At present there is no conclusive evidence to suggest that the flap shape affects surgical results.

### Intraoperative bleeding

Mild conjunctival bleeding during dissection is usually transient and stops spontaneously. Bleeding may occur more frequently in patients on oral anticoagulants, hypertensives and patients with increased capillary fragility. Significant subconjunctival hemorrhage may hamper visualization of scleral flap suture during argon suture lysis or may hinder bleb formation and may predispose to bleb failure.

Scleral bleeding in the scleral flap is usually controlled by direct pressure with a cotton-tipped applicator or irrigation with BSS. Cauterization may shrink the tissue and result in poor closure; in view of this cauterization should be avoided for scleral bleeding. During iridectomy or inner block removal injury to the major arterial circle of iris can cause bleeding. Irrigation or application of pressure stops the bleeding. If the bleeding persists one may have to close the flap and increase the IOP to stop the bleeding. Small hyphemas are usually self-limiting. Larger hyphemas require drainage via an AC paracentesis. AC bleeding was the most common peroperative complication (8%) in the collaborative initial glaucoma treatment study (CIGTS).[8,16]

Suprachoroidal hemorrhage (SCH) is a rare complication of glaucoma surgery. The risk factors for this complication are aphakia, vitrectomised eyes, congenital glaucoma, pathological myopia, patients on anticoagulants and significant hypotony.[17–19] Strong association of SCH with higher preoperative IOP[20] and longer axial length[21] has been reported. Preventive measures in high-risk groups include IOP reduction by medications including hyperosmotic agents and releasing aqueous gradually through the paracentesis tract. Preplacing scleral flap sutures facilitates closure of the scleral flap without resulting in significant duration of hypotony. The risk of SCH is greatly increased in patients with raised episcleral venous pressure like Sturge Weber syndrome and in dural sinus shunts. Preventive sclerotomies should be placed in high-risk eyes.[22] Progressive shallowing of the AC, loss of red reflex, onset of pain despite adequate local anesthesia and appearance of a dark posterior segment mass are signs of SCH. The scleral flap should be immediately closed and intravenous mannitol administered to lower the IOP. In select cases, sclerotomies are recommended to promote drainage of suprachoroidal fluid.

### Peroperative surgical pearls

1. Consider topical or parabulbar anesthesia
2. Never use toothed forceps to handle the conjunctiva
3. Aim for watertight conjunctival closure
4. Lower high preoperative IOP
5. Never leave the operating room with an unclosed leak

## Early Postoperative Complications

To a large extent, the outcome of the trabeculectomy surgery is determined by postoperative care. The frequency of visits varies according to the IOP, AC depth and bleb characteristics. During this period the patient should be instructed to avoid strenuous activity and advised to use topical steroids and cycloplegics. In each postoperative visit, the examination consists of measurement of IOP, AC depth and evaluation of bleb characteristics.

**Figure 1:**
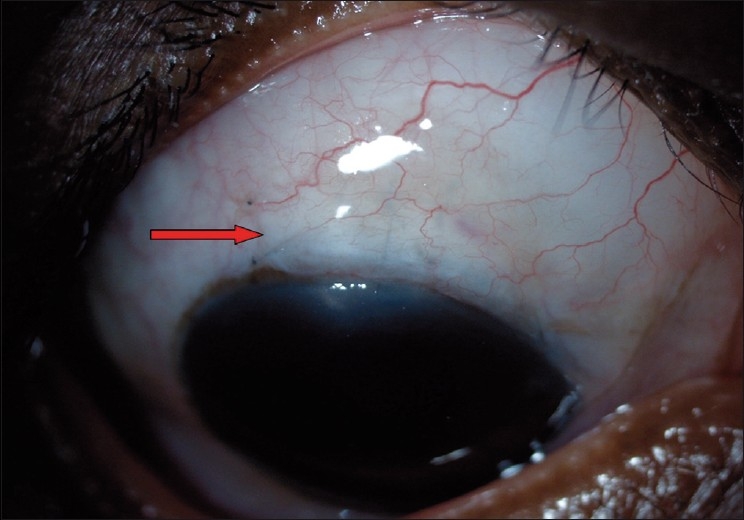
Diffuse bleb with reduced vascularization and cystic changes

A low-lying diffuse bleb with reduced vascularity, cystic changes, IOP in low teens, well-formed AC with tight conjunctival closure indicates an ideal bleb [Fig. 1]. Deviation from this picture suggests possibility of early postoperative problems.[23,24]

### High intraocular pressure with a deep anterior chamber

It is usually because of tight closure of the wound, however, one should perform gonioscopy if needed to rule out the obstruction to flow at the sclerotomy site. Obstruction is rare and is usually due to fibrin, blood, vitreous, iris or imperforate Descemet’s membrane. The obstruction with fibrin and blood is transient, whereas obstruction with iris or vitreous needs intervention.

Tight flap closure is the common cause for raised IOP with deep AC. The goal of management here is to separate the edges of the scleral flap with digital pressure.[25] This can be achieved by pressing on the sclera next to the flap with a cotton tip applicator or firmly compressing the globe with index finger over the lower eyelid while patient is looking upwards. The IOP, depth of the AC and height of bleb should be noted after the digital pressure. These measures may have to be repeated multiple times. If the IOP remains high, removal of the releasable suture or laser suturelysis can be considered. Releasable sutures placed at the time of surgery and their sequential release in the postoperative period is a useful technique. A variety of suturing techniques have been described.[26–28] These sutures provide tight closure of the wound in the immediate postoperative period and later allow IOP reduction with sequential release. It is mandatory to remove one suture at a time. Laser suturelysis is performed using the argon or diode laser. The initial technique was described in 1984[29] and since then has become a common practice in the postoperative management of trabeculectomy. Multiple instruments have been described for this procedure – the Hoskins stalk lens, Ritch, Mandelkorn and Blumenthal.[29–31] The common feature among these lenses is their ability to compress the conjunctiva and underlying tissue; this facilitates the visualization of the suture. Most of the sutures can be cut using low energy and low exposures.[32] Longer exposure can cause conjunctival coagulation and hole formation. Both techniques can be associated with complications such as flat anterior AC and wound leaks.[23] These methods are useful for a period of two to three weeks following trabeculectomy without antimetabolites. With adjunctive use of antimetabolites such as MMC, the window period for postoperative titration with suture removal is extended to several months; this timeframe is somewhat shorter with 5-FU.[23]

If the bleb remains flat with raised IOP in spite of digital massage and laser suturelysis, scarring at the episcleral surface is the cause for bleb failure. The failing bleb can be salvaged with 5-FU subconjunctival injections with or without needling of the bleb depending upon the extent of the scarring. The needling technique was originally described for encapsulated blebs, but was found to be very useful in failing blebs also.[33–35] The procedure can be easily done as an outpatient procedure under sterile conditions using a slit-lamp. Using a 30-gauge needle on a 1-ml syringe the subconjunctival space is entered away from the trabeculectomy site. The needle is passed subconjunctivally to the bleb site while visualizing the tip; a gentle sweeping motion is used to release all fibrosis. A course of subconjunctival injection of 5-FU (5 mg/0.1 ml) is then given, administering the injections away from the bleb site.[23]

**Figure 2:**
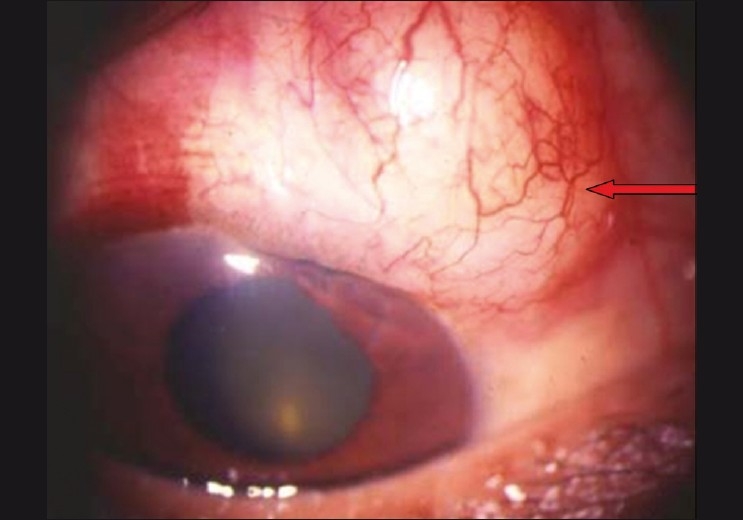
Encysted bleb—prominent blood vessels with thick wall

Another important cause for elevated IOP with a deep AC is bleb encapsulation [Fig. 2]. It is also referred to as Tenon’s cyst and it usually occurs during the second to fourth postoperative week as a tense, “tight-appearing” bleb. The bleb is firm with few or no microcysts. The IOP tends to rise with encapsulation but falls after two to four months. The reported incidence is 9–15% following trabeculectomy.[36–38] Temporary IOP reduction with medications such as aqueous suppressants is usually required. Bleb needling with antimetabolites is an option in case of sustained raised IOP. Failing all measures, a surgical bleb revision (partial/ complete cyst excision) or repeat trabeculectomy may be required, especially in cases of multiloculated cysts. A prospective study has shown superiority of medical therapy using the aqueous suppressants over needling of bleb in the management of encysted blebs.[39] Risk factors for encapsulation are use of limbal-based flap or long-term use of topical beta-blockers.[40,41]

### High intraocular pressure with shallow anterior chamber

Following trabeculectomy, shallow or flat AC can occur due to various reasons. Elevated IOP with shallow AC is mainly because of pupillary block, aqueous misdirection or suprachoroidal hemorrhage.

### Pupillary block

This should be considered first in the differential diagnosis of raised IOP with shallow AC. It may be difficult to differentiate from aqueous misdirection; however, the iris bombe caused by pupillary block usually is associated with a central AC deeper than the peripheral AC. The condition responds well to a laser or surgical iridectomy.

### Aqueous misdirection (Malignant glaucoma)

It is caused by posterior diversion and pooling of aqueous in the vitreous cavity. Sudden shallowing of the AC following conjunctival leaks, overfiltration following suture removal may be the initiating event of the cascade leading to misdirection of aqueous. This shallowing causes alteration in vitreous volume and its compaction leading to an increase in vitreous volume and reduced permeability of the aqueous through the anterior hyaloid.[42,43] This leads to progressive accumulation of aqueous in the vitreous cavity and uniform (central and peripheral) shallowing of the AC. Another recent hypothesis proposes that choroidal expansion contributes to the events causing anterior vitreal movement.[44]

**Figure 3:**
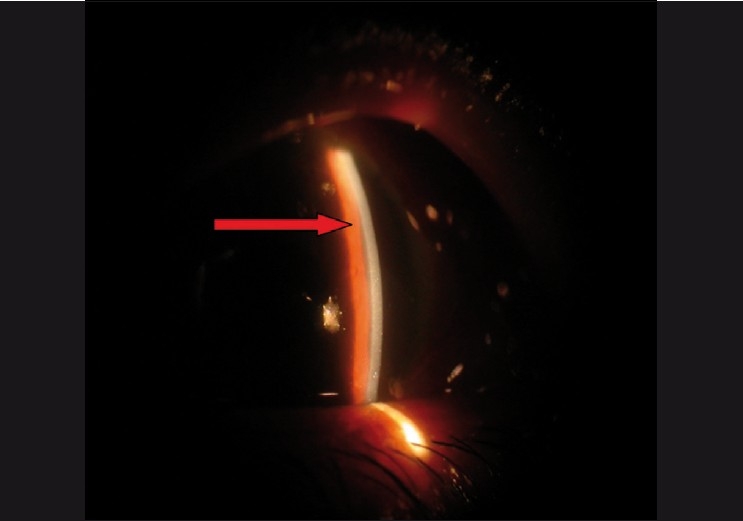
Aqueous misdirection with flat anterior chamber

**Figure 4:**
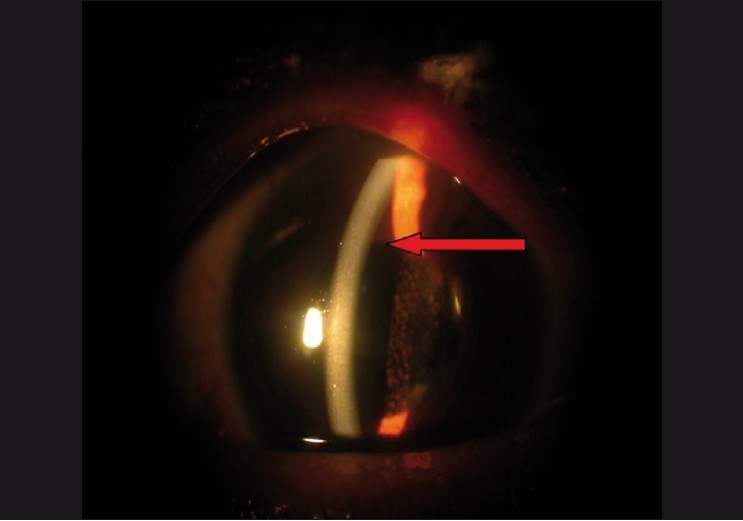
Responded to medical therapy, note the deep anterior chamber

Intraocular pressure may be normal to high. Initial management consists of aqueous suppressants and cycloplegics [Figs. 3 and 4]. If medical therapy fails, surgical treatment is required. In aphakic or pseudophakic eyes, the anterior hyaloid can be disrupted with the Nd: YAG laser in combination with posterior capsulotomy.[23] If the ciliary processes are visible they can be shrunk with the argon laser.[23] These measures can reverse the events and reestablish the normal aqueous flow and deepening of the AC. Incisional procedures may be needed in some cases. In the past, needle aspiration of the fluid vitreous was a commonly done procedure.[23] In the modern era it is preferable to do a pars plana vitrectomy with disruption of the anterior hyaloid face. The key to success here is establishing free flow of aqueous from the posterior chamber to the AC[45] (personal clinical experience). Intense surveillance is needed in these eyes because of possible relapse of the condition, especially in phakic eyes where disruption of the anterior hyaloid face was not sufficient. Long-term cycloplegic drops may be needed and it must be kept in mind that the fellow eye is at high risk of developing the same problem in case of any surgical intervention.

### Suprachoroidal hemorrhage

Delayed postoperative SCH is characterized by abrupt onset of pain, nausea and loss of visual acuity. Examination shows a peripheral and central flat AC, loss of red reflex and appearance of dark brown dome-shaped choroidal elevations. Ultrasonography demonstrates blood in the suprachoroidal space. Serial ultrasound examinations are needed to evaluate the size of the hemorrhage and also the liquefaction of the clot. Small hemorrhages can resolve spontaneously with topical and systemic steroids and larger ones will require drainage. Drainage is usually done once liquefaction occurs (usually two to four weeks) usually through an inferiorly placed sclerotomy with constant infusion of BSS in the AC.[23]

### Low intraocular pressure with shallow or flat anterior chamber and flat bleb

Usual causes for this postoperative condition are conjunctival wound leaks, serous choroidal detachments and rarely, an inadvertent cyclodialysis cleft.

### Conjunctival wound leaks

Hypotony without a visible bleb should alert the surgeon to the possibility of a conjunctival leak and the Seidel’s test usually localizes the site of the leak. The surface of the bleb and suture line should be tested for a leak. Management depends upon the size and position of the leak, the appearance of the bleb, depth of the AC and whether antimetabolites were used or not. Usually, in eyes with a deep AC and a well-formed bleb, a small leak along the suture line heals well, either spontaneously or with conservative treatment. In eyes that receive antimetabolites, even small leaks may not heal and surgical closure may be needed. Large leaks along the suture line need closure. Surface leaks are difficult to close surgically and conservative treatment should be tried. Conservative treatment consists of patching of the eye, aqueous suppressants and the use of antibiotic drops known to induce scarring such as gentamycin or tobramycin. If there is no response, various techniques have been described–bandage contact lens,[46] collagen shield,[47] tamponade with Simmons shell[48] or symblepharon ring, tissue adhesives such as cyanoacrylate[49] or fibrin glue,[50] and autologous blood injection.[51] In eyes that fail to respond, one may have to resort to surgical correction. The best way to avoid postoperative leaks is to meticulously close the conjunctiva peroperatively. It is strongly recommended to look for any leaks at the conclusion of the trabeculectomy and if there are any leaks they should be handled with appropriate measures.

### Serous choroidal detachment

The precipitating factor for the serous choroidal detachment is usually hypotony. The detachments may further reduce aqueous flow and start a vicious cycle. Most cases usually resolve on conservative therapy that consists of frequent topical steroids and cycloplegics with or without systemic steroids along with management of the event precipitating hypotony. One should rule out inadvertent use of aqueous suppressants also.[52] If all measures fail, surgical intervention is necessary, especially in cases of cornea-lenticular touch or large non-resolving effusions. Choroidal drainage is usually done under general or local anesthesia. The eye is rotated upwards using an inferior corneal or rectus traction suture. Infero-nasal or infero-temporal quadrants can be chosen for drainage, depending on the most dependent area of detachment. After incising the conjunctiva and Tenon’s layer in a radial manner about 5-6 mm away from the limbus, a radial scleral incision is made and slowly dissected to reach the suprachoroidal space. Escape of straw-colored fluid is seen on reaching the suprachoroidal space. During the drainage, AC deepening with BSS or viscoelastic should be done. An AC maintainer can also be used. Pressure on sclera near sclerotomy and passing a spatula between the sclera and the choroid through the sclerotomy facilitates drainages. Gentle cautery to edges of the sclerotomy helps in keeping it open allowing a continuous drainage of fluid. The conjunctiva and Tenon’s layers are closed in layers. It may take several months for the complete resolution of detachment and cataract formation is commonly seen.[53]

### Low intraocular pressure with shallow or flat anterior chamber and elevated bleb

Excessive filtration (over-filtration) due to a loose scleral flap suture is the common cause for this postoperative complication. The condition usually resolves spontaneously. Treatment is recommended when the AC is very shallow or if hypotony is associated with large choroidal effusions. Aggressive cycloplegia with 1% atropine and pressure patching, with judicious use of aqueous suppressants to reduce the excess aqueous outflow, helps to deepen the AC. In non-responding cases surgical intervention may be needed. Deepening the AC with viscoelastic, air, or non-expansile concentrations of gas may be sufficient in some cases.[23] Large choroidal effusions may need drainage. Persistent early hypotony can lead to chronic hypotony. Aggressive laser suturelysis or release of releasable sutures can also cause this postoperative complication.

### Early postoperative period - Points to ponder

1. Digital massage helps in formation of the bleb
2. Laser suturelysis and releasable sutures are useful techniques, tackle one suture at a time to avoid hypotony, especially in eyes that received antimetabolites
3. Early recognition of signs of scarring and appropriate treatment improves the success rate of trabeculectomy
4. Always try conservative treatment with encapsulated blebs
5. Aqueous misdirection is a difficult complication to manage; the best approach is to prevent the initiating factor (sudden shallowing of the AC) in high-risk eyes.
6. Common cause for serous choroidal detachment is a conjunctival wound leak. Identification of primary cause and appropriate treatment should be the first step in the management.

## Late Postoperative Complications

Late postoperative complications following trabeculectomy are mainly due to the long-term changes in the bleb characteristics. With the advent of antifibrotic agents such as 5-FU or MMC we are able to achieve lower target IOPs. However, late complications such as chronic hypotony, bleb leaks, blebitis and endophthalmitis have increased. Under this section we will be discussing some of the important late complications of trabeculectomy.

### Chronic hypotony

**Figure 5:**
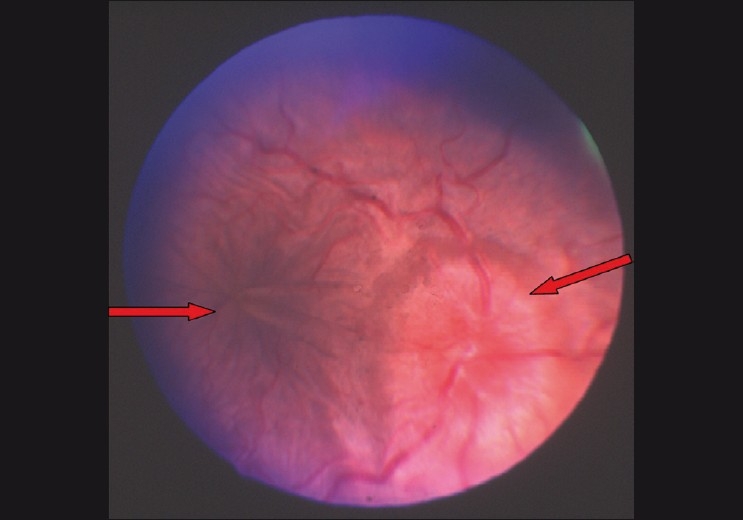
Hypotony maculopathy: Disc edema with macular folds

When hypotony (IOP of less than 5 mmHg) persists for more than three months it is called chronic hypotony. This can be associated with a drop in the visual acuity[54] and hypotony maculopathy. Maculopathy is characterized by choroidal folds, retinal striae and no edema[55] [Fig. 5]. Reported risk factors for this complication are young age and myopia.[56,57] These factors are mostly related to decreased scleral rigidity in the area of the posterior pole and a tendency towards collapse in the presence of low IOP. Reported non-surgical interventions can be tried before resorting to the surgical revision of the bleb. Commonly used interventions are–soft contact lenses, bleb size reduction by cryotherapy,[58] autologous blood injection with or without compression sutures,[59–61] and argon laser to the bleb.[62] Most of the times the results are inconsistent with these modalities. Surgical revision consists of closing the scleral flap or applying a scleral patch graft in cases of scleral dehiscence; this results in increasing the IOP and restoring the visual function.[57,63] Avoidance of hypotony altogether with primary surgery is the optimal procedure to avoid this long-term complication.[64]

### Leaking blebs

**Figure 6:**
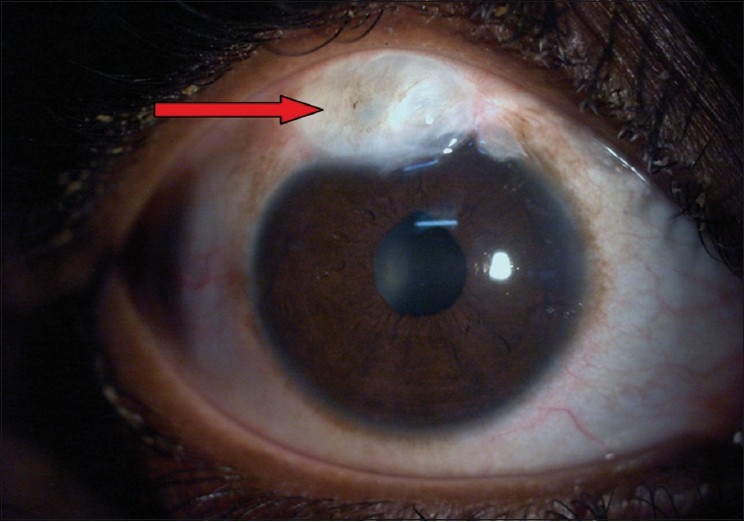
Cystic bleb with a bleb leak

**Figure 7:**
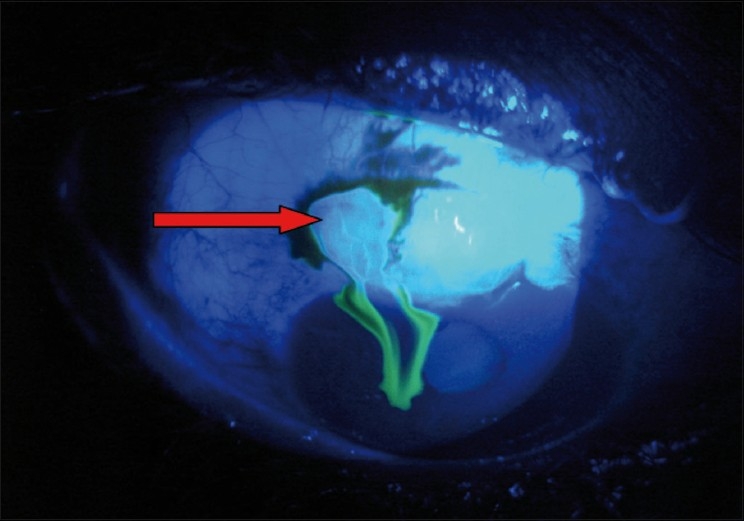
Showing positive Seidel’s test

**Figure 8–10:**
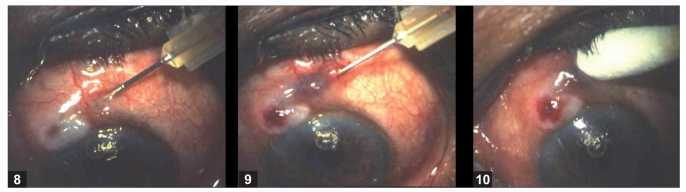
Technique of autologous blood injection in a patient with bleb leak

They are usually due to the thin-walled blebs that are common with antimetabolite-supplemented trabeculectomies.[65] The frequency of the late bleb leaks ranges from 1.8–10% and the FFSS Group reported a 7% rate after three and five years’ follow-up.[66] Bleb leaks are detected with Seidel’s test: the fluorescein strip is applied gently over the bleb and the eye is examined using cobalt blue illumination. If there is leak, unstained aqueous will be seen to flow surrounded by dark green fluorescein tear film [Figs. 6 and 7]. In the absence of spontaneous leak, gentle pressure can be applied and suspicious area of leak should be examined. A small hole may appear in thin-walled blebs, causing leak and inflammation. In blebs without antimetabolite use, these leaks can subside with conservative treatment which consists mainly of aqueous suppressants, broad-spectrum antibiotics and patching or soft contact lens application. During this period the patient should be instructed to watch for any symptoms of endophthalmitis. If the leak is large or not responding to conservative treatment, other modalities that result in closure of the leaks should be tried. Reported modalities are cyanoacrylate glue,[49] fibrin tissue glue,[50] injection of autologous blood[51] and surgical revision.[52,66] We perform the autologous blood injection procedure in the operation theater to maintain good asepsis [Figs. 8–10]. As no anticoagulant is used, the procedure must be done quickly. After cleaning the antecubital region, the assistant withdraws blood into a 1-ml syringe and the surgeon changes the needle to 30-gauge and introduces the needle at least 5 mm away from the bleb. The needle is observed entering the bleb, where 0.1-0.3 ml of blood is injected. Some blood may enter the AC during the procedure, which can be washed via a paracentesis if required. When simple methods fail or the leak is complicated, surgical revision is recommended. The ideal treatment would eliminate the leak as well as hypotony while maintaining the filtration function of the bleb. We use a free autologous conjunctival patch graft and the procedure can usually be done under peribulbar anesthesia.[67] Superior and inferior rectus traction sutures are taken using 6-0 silk. A paracentesis is performed and the AC is formed with an air bubble or with viscoelastic, if necessary. The avascular necrotic area of the bleb is excised at the junction of avascular and vascular conjunctiva using Wescott’s scissors. The anterior extent of the dissection is carried out to the limbus. The underlying sclera is inspected. Additional sutures are applied to the scleral flap wherever necessary. A scleral patch graft is used to repair areas of necrotic sclera wherever necessary. The conjunctival defect is measured with calipers. The cornea anterior to the bleb is denuded of epithelium and a shallow groove is fashioned 1 mm anterior to the limbus to anchor the graft to this region. An area about 1 mm in dimension, larger than the bed, is marked with a marking pen in the inferior bulbar conjunctiva, adjacent to the limbus. The demarcated area is excised taking care to include the underlying Tenon’s capsule. The fornix is avoided to prevent foreshortening. This is then placed on the scleral bed taking care to maintain limbal orientation. The conjunctival patch graft is secured to the donor site using 10-0 nylon sutures. The four corners are sutured first and then the remainder of the graft is secured using continuous interlocking sutures. The limbal edge of the graft is anchored to the shallow corneal groove created earlier. Antibiotics, steroid and cycloplegic drops are administered postoperatively.

### Symptomatic blebs

**Figures 11, 12:**
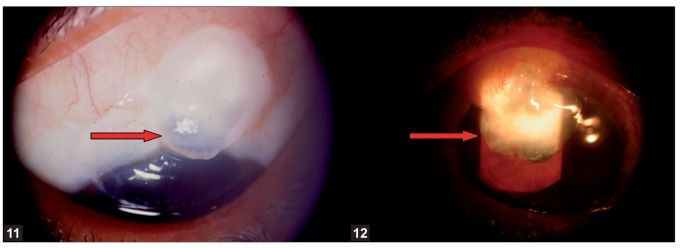
Symptomatic blebs—localized thin cystic bleb and overhanging bleb

**Figure 13:**
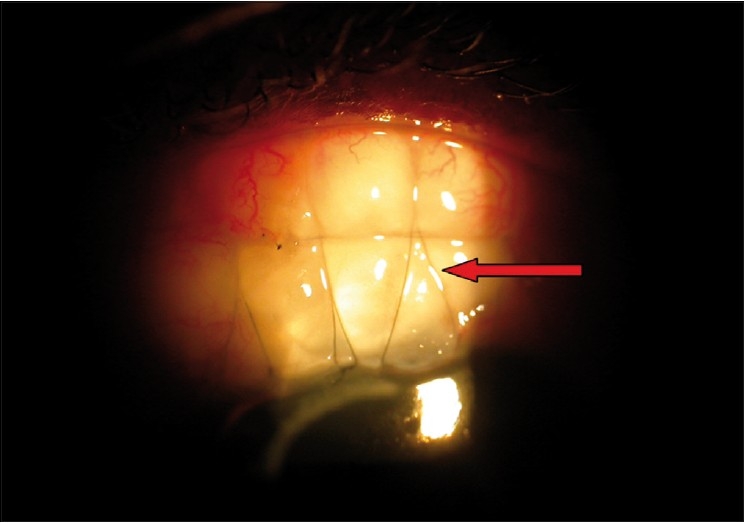
Compression sutures in place for overfiltering bleb

The filtering blebs are reasonably tolerated and most patients are aware of “blister” and some report various degrees of discomfort. Symptoms are common with nasal or large blebs or blebs extending to the cornea [Figs. 11 and 12].[66] Symptoms can be associated with superficial punctate keratopathy, tear film abnormalities, dellen formation and ocular surface irregularities. Symptoms are mainly foreign body sensation and visual disturbances. Frequent use of artificial tears and ocular lubricants are recommended as initial treatment. If the symptoms persist, surgery should be considered for large blebs or overhanging blebs.[66] Compression sutures can be used to reduce the height of the large elevated bleb. Compression sutures are placed using 9-0 or 10-0 nylon suture on a cutting needle. A partial thickness corneal pass is made anterior to the bleb. A second pass is made posterior to the bleb after incising the conjunctiva and episclera. The suture is tied in an X over the bleb, so as to compress the bleb surface towards the sclera. More than one suture can be placed. The technique also appears to cause a remodeling within the bleb [Fig. 13].

### Failing or failed blebs

Late failure of the blebs is mainly due to the fibrosis at the conjunctival and episcleral interface with a patent sclerotomy and rarely due to obstructed sclerotomy. Management depends upon the cause. External revision with needling using antimetabolites is the commonly recommended procedure for bleb failure due to scarring. Internal revision with laser can be tried for sclerotomy obstruction. When these measures fail, repeat glaucoma surgery will be needed.[66]

### Bleb-related ocular infections

**Figure 14:**
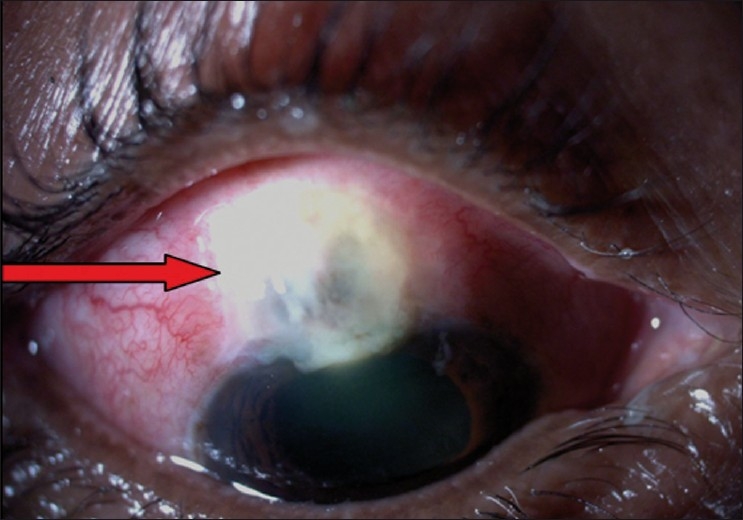
Blebitis (exudates in bleb seen)

**Figure 15:**
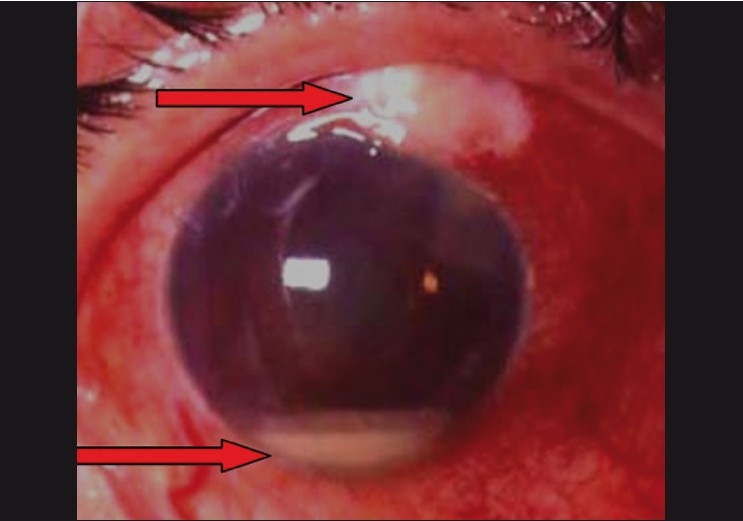
Blebitis with endophthalmitis causing a hypopyon

The presence of thin-walled blebs that is commonly seen with antimetabolite use is a risk factor for bleb infections. The infection usually starts in the subconjunctival space and spreads to the AC and the vitreous cavity. Onset of infection can vary from the first few days post surgery to 20 years following filtration surgery.[68] Risk factors for the infection include thin-walled blebs with leaks, myopia, the presence of releasable sutures, concurrent respiratory infections, blebs located at the inferior limbus, unguarded filtration surgery and diabetes mellitus.[68–71] The reported incidence of bleb-related endophthalmitis is as high as 2%, even higher estimates have been reported of 6% of blebitis and 7.5% of endophthalmitis.[72] The morbidity of infections can be very high, almost one-third of bacterial infections following filtering surgery that were treated with intensive medical treatment ended up with no light perception at the end of one year. Positive bacterial cultures carried a worse visual prognosis.[73] Certain organisms can spread through the conjunctiva.[66] The lens and an intact posterior capsule with a posterior chamber intraocular lens are relative barriers to migration of bacteria but not absolute barriers.[66] The source of bacteria is usually ocular flora and the most common organisms are the Streptococcus species, Staphylococcus species and *Haemophilus influenzae*.[66] There may be regional differences in types of bacterial infections. Common symptoms are ocular pain, foreign body sensation, blurred vision and tearing with or without a history of red eye with discharge. On examination, the bleb typically will have a milky white appearance with loss of clarity. It may be associated with bleb leak, hypopyon and vitreous reaction [Figs. 14 and 15]. It can be classified as Stage I - bleb involved, Stage II - AC involved (Stage I+ AC reaction) and Stage III - Vitreous involved (Stage II + vitreous reaction).[66] Stage I (blebitis) is likely to respond to intensive antibiotic treatment with more favorable outcome. When there is involvement of the AC and the vitreous cavity, a fluid tap should be taken and sent for microbiological examination. Topical and systemic antibiotics will be needed. Full-blown endophthalmitis will need aggressive treatment in the form of intravitreal injections and vitrectomy. It is best managed in consultation with a retinal specialist. The role of prophylactic use of topical antibiotics to prevent bleb-related infections is questionable.[66] Any conjunctivitis and blepharitis should be treated promptly. Patients should be educated about early symptoms of infections. Bleb leaks are the predisposing factors for infection, clinicians should look for the leaks during follow-up visits and treat them immediately if present. Patients should be warned not to rub the eyes with blebs and not to swim in water likely to be contaminated.

### Late postoperative complications - Points to remember

1. Early postoperative hypotony leads to chronic hypotony. Avoid low IOP, especially in eyes treated with antimetabolites
2. Patient education is important to minimize the risk of bleb leak and infections related to it
3. Failure of the bleb can occur any time following the surgery, post surgery follow-up is a must for all patients
4. Tear film disturbances are a common cause for symptomatic blebs and need artificial tears
5. Immediate and aggressive treatment of bleb infections can salvage the eye

To conclude, in filtering surgeries, postoperative management may at times be more challenging than the procedure itself. Good preoperative evaluation, meticulous intraoperative technique and appropriate postoperative management are mandatory for all cases for a successful outcome. Long-term monitoring of the trabeculectomy blebs is needed to minimize the late complications.

## Complications of Non-penetrating Glaucoma Surgery

The popular surgeries in this group are viscocanalostomy and deep sclerectomy. The principle of both surgeries appears to be different; however, there is lot of overlap in the surgical technique. In viscocanalostomy the emphasis is on dilating the Schlemm’s canal while in deep sclerectomy the focus is on deroofing the Schlemm’s canal to create an intrascleral reservoir. The advantages of non-penetrating surgery over standard trabeculectomy are in the avoidance of sudden decompression of the eye, less chances of hypotony, intraocular inflammation, intraocular bleeding and fewer postoperative visits with more rapid visual rehabilitation. The disadvantages are that it is technically more difficult to perform, costs more and may not result in low enough IOP. In addition, IOP control may not last long.[6] The main complications of viscocanalostomy are the inability to find Schlemm’s canal, micro perforations or full-thickness perforation into the AC resulting in conversion to trabeculectomy. Deep sclerectomy is performed more often than viscocanalostomy. The complications of deep sclerectomy include perforation leading to conversion to trabeculectomy, scleral ectasia, iris incarceration, Descemet’s detachment and late AC bleeding during gonioscopy. The need to perform postoperative Nd:YAG laser goniopuncture in a significant proportion of cases for better control of IOP is another limitation. The reported incidence of laser goniopuncture varies from 3–80%, intraoperative use of MMC seems to reduce this incidence. Laser goniopuncture can cause iris incarceration resulting in raised IOP.[74,75] Long-term data on these procedures are limited. The American Academy of Ophthalmology prepared an ophthalmic technology assessment on non-penetrating glaucoma surgery in 2001.[76] Based upon 100 publications they reported that both surgical procedures lower IOP to mid or upper teens with a low complication rate related to overfiltration or hypotony. However they highlighted the need for more randomized controlled studies to clarify a lot of questions associated with these procedures.

## Complications of Drainage Devices

In general, drainage devices are reserved for eyes with a lot of conjunctival scarring where conventional trabeculectomy is likely to fail. They rarely deliver the low IOP that is needed in advanced glaucomas. At the end of one year 60-80% maintain IOP of less than 22 mmHg.[77,78] Postoperative hypotony should be avoided either by using a valved device or ligating the tube. Motility problems have been reported, especially with Baerveldt implants. This is due to mechanical involvement of superior oblique or an elevated bleb causing limitation of muscle movement. The ocular hypertensive phase usually occurs one to three months following the surgery. This phenomenon is due to capsular fibrosis and is commonly seen with a single sitting procedure with early contact between aqueous and the conjunctiva. This condition may be difficult to differentiate from a steroid-induced response. Treatment involves aqueous suppressants and topical steroids. Tube and plate migration can occur as late complications. Tube migration can be corrected with tube extenders and migrating plate can be resutured again. Partial or complete exposure of the device is difficult to treat and may necessitate removal of the device. The tube versus trabeculectomy (TVT) study is a multicenter randomized clinical trial, designed to compare the safety and efficacy of tube shunt surgery to trabeculectomy with MMC in patients with previous cataract and/or glaucoma surgery.[7] They reported higher incidence of complications in the trabeculectomy group, however, all complications were not equal in severity and the rate of serious complications was equal in both groups. Long-term complications of incisional glaucoma surgery were evaluated among Medicare beneficiaries. In this report they found that the complication rates were significantly greater in the glaucoma drainage device group.[79]
